# Clinical and Immunovirological Characteristics Associated with Cardiovascular Dysautonomia in Long COVID

**DOI:** 10.3390/jcm15114192

**Published:** 2026-05-28

**Authors:** Yves Renaudineau, Selena Teillaud, Sébastien De Almeida Chaves, Muriel Alvarez, Romain Barthes, Chloé Bost, Françoise Fortenfant, Bénédicte Puissant-Lubrano, Florence Abravanel, Camille Vellas, Anne Pavy-Le Traon, Laurent Sailler

**Affiliations:** 1 Laboratory of Immunology, Referral Medical Biology Laboratory, Institut Fédératif de Biologie, Toulouse University Hospital Center, 31300 Toulouse, France; renaudineau.y@chu-toulouse.fr (Y.R.); selena.teillaud@etu.unilim.fr (S.T.); bost.c@chu-toulouse.fr (C.B.); fortenfant.f@chu-toulouse.fr (F.F.); puissant.b@chu-toulouse.fr (B.P.-L.); 2 Toulouse Institute for Infectious and Inflammatory Diseases (INFINITy), INSERM U1291, CNRS U5051, University of Toulouse, 31062 Toulouse, France; 3 Department of Internal Medicine, CHU Toulouse, 31059 Toulouse, France; dealmeida.se@chu-toulouse.fr; 4 Department of Infectious Diseases, CHU Toulouse, 31059 Toulouse, France; alvarez.m@chu-toulouse.fr; 5 Department of Pneumology, CHU Toulouse, 31059 Toulouse, France; barthes.rom@chu-toulouse.fr; 6 Laboratory of Virology, Institut Fédératif de Biologie, CHU Toulouse, 31300 Toulouse, France; abravanel.f@chu-toulouse.fr (F.A.); vellas.c@chu-toulouse.fr (C.V.); 7 Department of Neurology, Toulouse University Hospital Center, I2MC-INSERM 1297, 31059 Toulouse, France; pavy-letraon.a@chu-toulouse.fr

**Keywords:** long COVID, cardiovascular dysautonomia, Postural Tachycardia Syndrome, age, obesity, anti-viral, inflammation, allergy, autoimmunity

## Abstract

**Background/Objectives:** This report is an assessment of the characteristics associated with cardiovascular dysautonomia (CVD) in the context of long Coronavirus disease (COVID), which is currently inadequately characterized. **Material and Methods:** A retrospective cross-sectional study was performed involving 106 patients with long COVID, including 34 individuals diagnosed with CVD, among whom eight met the criteria for Postural Tachycardia Syndrome (PoTS). The variables assessed encompassed individual characteristics (e.g., age, sex, comorbidities), immunization parameters (e.g., vaccination/viral status, timing, frequency), cellular and humoral anti-Spike and anti-Nucleocapsid (Nuc) immune responses, inflammatory and allergic biomarkers, as well as an extensive panel of common autoantibodies comprising anti-nuclear antibodies, anti-central nervous system antibodies (cerebellum, brain), and anti-peripheral nervous system antibodies (gangliosides). **Results:** An age < 45 years, body mass index, hyperventilation syndrome as well as a higher cumulative number of antigenic contacts (vaccinations plus infections ≥ 3) and an elevated basophil count (≥0.06 G/L) were independently associated with CVD. There was no association between CVD and inflammatory markers or common autoantibodies. Patients with PoTS criteria had a strong anti-Spike cellular immune response and increased IgG anti-Nuc humoral immunity when compared with CVD and non-CVD long COVID counterparts. **Conclusions:** Compared to other long COVID patients, patients with long COVID-associated CVD have distinctive clinical and immunovirological features. Our results suggest the potential role of the immune response against Spike and of allergic pathways rather than humoral autoimmunity against common autoantibodies in long COVID CVD.

## 1. Introduction

A SARS-CoV-2 infection can result in long-term cardiovascular dysautonomia (CVD) characterized by abnormal autonomic reflexes upon standing, with a subset meeting diagnostic criteria for Postural Tachycardia Syndrome (PoTS) [1]. CVD develops during or following an acute COVID-19 infection, persisting beyond three months to years after exclusion of alternative diagnoses [2]. Among patients experiencing long COVID, the prevalence of CVD ranges from 10 to 30%, and exhibits significant variability in severity and duration among individuals [3]. Specific risk factors for CVD-associated long COVID are largely unknown as compared to long COVID in general, which is associated with a younger age, female sex, obesity, various comorbidity factors, and lack of anti-COVID vaccination [4].

The etiopathogenesis of long COVID-related CVD and PoTS remains under debate, with evidence supporting inflammatory, allergic, autoimmunity, viral persistence, and a circulating spike protein effect as potential contributors [5]. Discrepancies among findings are largely attributable to the observational nature of existing studies, their focus on isolated etiologic factors, and comparison with healthy controls instead of non-CVD long COVID counterparts. The present paper is dedicated to describing the clinical and immunological characteristics of patients with post-COVID CVD compared with patients with long COVID without CVD.

## 2. Materials and Methods

### 2.1. Patients

We made a retrospective cross-sectional study from March 2022 to June 2025. A hundred and six patients with developed long COVID were recruited at the Department of Internal Medicine of Toulouse University Medical School. Long COVID was established for more than 3 months post-acute and RT-PCR/antigenic proven SARS-CoV-2 infection and after excluding any alternate diagnosis. Among them, the diagnosis of CVD-associated long COVID was assessed in patients who (i) were symptomatic, reporting palpitations, malaise, inappropriate tachycardia relative to exertion, and/or exercise or orthostatic intolerance; and (ii) exhibited a resting heart rate exceeding 100 beats per minute (bpm) in the supine position, orthostatic hypotension, or PoTS (+30 bpm without associated hypotension within 10 min of standing not attributable to other identified factors) [5]. Patients whose treatment precluded the assessment of CVD presence were excluded, as were those presenting with alternative potential causes of dysautonomia. Hyperventilation syndrome (HVS) was suspected when the Nijmegen score exceeded 23 and confirmed through specific investigations involving respiratory function tests [5]. Information collected from medical records included demographic data (age, sex), information on SARS-CoV-2 infections and vaccinations (number, time from last immunization against the virus or the vaccine), comorbidities (obesity, allergy, and HVS), and routine inflammatory and allergy biomarker parameters (C-reactive protein [CRP], neutrophils to lymphocytes ratio [NLR], basophil blood count, tryptase). The cellular and humoral response against coronavirus and autoimmunity features were also systematically assessed as described below.

### 2.2. SARS-CoV-2 Assays

To test T cell response against SARS-CoV-2 Spike and Nucleocapside recombinant and endotoxin-free Spike and Nuc proteins (Invivogen^®^, Toulouse, France), 1 mL of whole blood was distributed in 4 heparinized tubes with: (i) 20 μL of SARS-CoV-2 full-length Spike protein (2 μg/tube); (ii) 2 μL of SARS-CoV-2 Nuc protein (2 μg/tube); (iii) 20 μL of RMPI (negative control); and (iv) 20 μL of phytohemagglutinin (PHA, 40 μg/mL). After 18–24 h incubation at 37 °C, tubes were centrifuged, supernatant concentration of IFN-γ quantified (Qiagen, Hilden, Germany), and results are expressed as international units (IU) of IFN-γ/mL (2 × 10^4^ IU/µg IFN-γ). For analysis, the value from the negative control tube was subtracted from the signal obtained after stimulation with recombinant proteins. IGRA-Spike and IGRA-Nuc thresholds for positivity were fixed at 0.040 IU IFN-γ/mL as previously described [6,7,8]. The test is recorded as indeterminate when the negative control is >8 IU IFN-γ/mL or when the mitogen control is <0.5 IU IFN-γ/mL, but such cases were not observed in this study.

The serological tests were carried out on serum and the level of IgG antibodies to SARS-CoV-2. Spike mammalian cell-expressed recombinant protein was assessed by using the SARS-CoV-2 IgG II Quant assay (Abbott Laboratories, Chicago, IL, USA). ELISA total values are expressed in BAU/mL, and with an assigned cutoff at 7.14 BAU/mL, as previously described [9]. The SARS-CoV-2 IgG assay (Abbott Nuc, Chicago, IL, USA) was used to detect anti-Nuc antibodies using a threshold fixed at 1.4 [10].

### 2.3. Other Immunological Data

Antibodies targeting central nervous system (CNS) (cerebellum, brain) and peripheral nervous system (PNS) (gangliosides) antigens, as well as nuclear antigens (ACAN on HEp-2 cells, extractable nuclear antigens [ENA], dsDNA/chromatin, cardiolipin/β2 glycoprotein I [CL/β2GPI]), were determined through indirect immunofluorescence (Kallestad HEp-2 cells, Bio-Rad, Hercules, CA, USA) and immunoassays (Bioplex2000, Bio-Rad) [11]. Antibodies against the central nervous system (cerebellum, brain from primate) were assessed by indirect immunofluorescence (Nova lite, Werfen, Barcellona, Spain) and followed by immunodot when positive (Orgentec Diagnostika GmBH PNS11, Mainz, Germany), antibodies against peripheral nervous system (gangliosides) antigens were assessed by immunodots (Generic assays Ref5003, Abacus dx Pty Ltd., Cannon Hill, QLD, Australia). CNS and PNS auto-antibodies detected by the immunodots are directed towards GAD65, Zic4, Tr(DNER), SOx1, Ma2, Ma1, Amphiphysine, CV2, Ri, Yo, HuD (for CNS) and GM1, GM2, GM3, GM4, GD1a, GD1b, GD2, GD3, GT1a, GT1b, GQ1b and sulfatides (for PNS). Pro-inflammatory cytokines (IL-1β, TNF-α, IL-6, IL-8; ELLA, Bio-Techne, Minneapolis, MN, USA) and tryptase levels (UNICAP250, ThermoFisher Scientific, Asheville, NC, USA) were quantified by immunoassay.

### 2.4. Statistical Analysis

Quantitative data are presented as median and interquartile (IQ) at the 25th–75th percentile or mean (±SD) and analyzed using non-parametric tests with Dunn’s test applied for *post hoc* multiple comparisons when necessary. For the multivariable logistic regression analysis, due to a low events-per-variable (EPV = 6.8) and in order to control the risk of overfitting, we used a LASSO penalized logistic regression model with cross-validation to optimize the penalty parameter. Non-zero coefficients were interpreted as predictors associated with the outcome. Variables validated by the LASSO regression were included in a classical logistic multivariable regression to present non-biased regression parameters and odds ratios (OR). We then performed a sensitivity excluding patients with POTS from the dataset because the pathophysiology of POTS may be different from that of other CVD subsets. The analysis used a LASSO regression for the selection of variables followed by a multivariable logistic regression.

The alpha risk was set to 0.05 if unspecified and statistical analysis performed using GraphPad Prism 10.2 (La Jolla, CA, USA) and SAS OnDemand for Academics, version SAS Studio 9.4 (https://www.sas.com).

## 3. Results

### 3.1. Long COVID Demographic Data (Figure 1)

**Figure 1:**
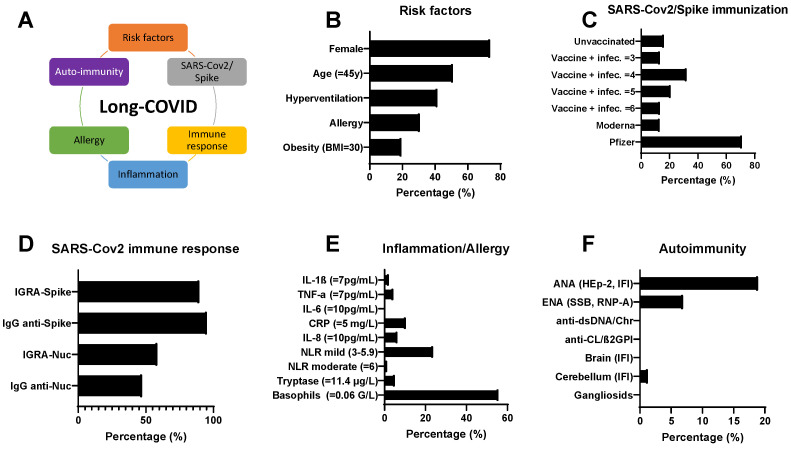
Variables assessed in 106 patients with long COVID (**A**). (**B**) Individual risk factors, BMI: body mass index. (**C**) SARS-CoV-2 infection (infec.) and vaccine (Spike) immunization parameters. (**D**) Cellular and humoral anti-Spike and anti-Nucleocapsid (Nuc) immune responses. The interferon gamma release assay (IGRA) was used to assessed cellular response. (**E**) Inflammatory and allergic biomarkers including the Neutrophil to Lymphocyte ratio (NLR). (**F**) Autoantibodies comprising anti-nuclear antibodies (ANA, HEp-2), anti-extractable nuclear (ENA) antibodies, anti-dsDNA/Chromatin antibodies, anti-cardiolipin/anti-β2 GPI antibodies, anti-central nervous system antibodies (cerebellum, brain), and anti-peripheral nervous system antibodies (gangliosides). Indirect immunofluorescence assays (IFI) are indicated.

We included 106 patients (female: 74%). Mean age was 45 (±12.6) years. Median time since first and last identified antigenic contact was 802 [549–1080] and 221 [IQ: 100–480] days, respectively. HVS was present in 44/106 patients (41.5%), allergy in 32/106 (30.2%), obesity defined by a BMI ≥ 30 in 17/106 (18%) (Figure 1B). At the time of consultation, 16% of patients had developed long COVID without prior vaccination; among those vaccinated, the Pfizer vaccine was predominantly administered (71%) as reported in France (https://covidtracker.fr/vaccintracker/ accessed on 30 October 2025). The median number of antigenic exposures, comprising both reported infections and vaccinations, was four [IQ: 3–5], spanning from one to eight contacts overall (Figure 1C). Patients retained cellular and humoral immunity against the Spike protein in 89.6% and 95.3% of cases, respectively, and against the nucleocapsid protein in 58.3% and 47.2%, respectively (Figure 1D).

An evaluation using an extensive panel of biomarkers for inflammation indicates that inflammatory status was absent or mild (Figure 1E). Regarding NLR, a mild NLR status ranging from 3 to 5.9 was observed in 23.7% of patients and in one case NLR was high (71.5) due to lymphopenia. A CRP level over 5 mg/L was detected in 10.4% (in positive: median 8.8 mg/L [IQ: 6–10]). Median basophil count was 0.06 [0–0.1] and 47/106 (44.3%) patients were above normal range (normal reference value < 0.1 G/L). A positive serum tryptase assay over 11.4 µg/L was identified in 5% of the cohort (in positive: median 13 µg/L [IQ: 12–16]) and only one of these patients had CVD (Figure 1E). Concerning autoimmunity profiles, a speckled, nonspecific antinuclear antibody pattern on HEp-2 cells was present in 18.9% of cases at low dilutions (1:160 to 1:360); this finding was deemed non-pathogenic given the absence of ENA antibodies, anti-dsDNA/chromatin antibodies, and anti-CL/β2GPI antibodies, except for four patients who exhibited low levels of either isolated anti-SSB antibodies (one case) or anti-U1 snRNP antibodies (three cases) (Figure 1F). Concerning autoantibodies targeting the CNS and the PNS, all findings were negative except for one patient who exhibited a mild, uncharacterized IgG binding within the molecular layer of the cerebellum.

### 3.2. Factors Associated with CVD

The diagnosis of CVD was confirmed in 34 patients. Univariate logistic regression analysis (Table 1) allowed the selection of variables associated with CVD risk at *p* < 0.20. Those were a lower BMI (*p* = 0.02), younger age (<45 years; *p* = 0.04), presence of an HVS (*p* = 0.10), an allergic background (*p* = 0.12), a longer duration (>1 year) since last infection and/or vaccine antigenic contact (*p* = 0.02), having three or more identified immunization contacts (vaccinations and/or infections combined) (*p* = 0.12) and a basophil count ≥ 0.06 G/L (OR 2.09 [0.89–4.91], *p* = 0.09). Of note, when analyzed individually, the number of infections and vaccinations was not associated with CVD, and the number of identified contacts with the Spike antigen was not related to time elapsed since first antigenic contact (Spearman correlation coefficient = 0.12; *p* = 0.21).

Among variables associated with CVD at a *p*-value < 0.20 in the univariate regression analysis, the LASSO model identified age < 45, BMI, hyperventilation syndrome, basophil count and number of immune contacts ≥3 as relevant for the multivariable logistic regression. Independent variables associated with CVD were a younger age (<45 years; odds ratio [OR] 2.9, 95% CI: 1.09–7.77), presence of HVS (OR 2.9, 95% CI: 1.10–7.55), total number of antigenic contacts ≥ 3 (OR 4.1, 95% CI: 1.10–14.9), and basophil count ≥ 0.06 G/L (OR 3.26, 95% CI: 1.9–8.8). Conversely, BMI was inversely related to CVD risk (OR 0.87, 95% CI: 0.79–0.97).

For the sensitivity analysis, The LASSO model identified the same variables as those identified in the main analysis as being relevant. The best multivariate logistic regression model (lowest AIC and the best classification of observations) included all these variables (Table 2). Overall, the ORs remained very close to the ORs calculated in the main analysis, though some variables no longer reached the 0.05 level of statistical significance due to loss of power.

### 3.3. Factors Associated with PoTS

**Figure 2:**
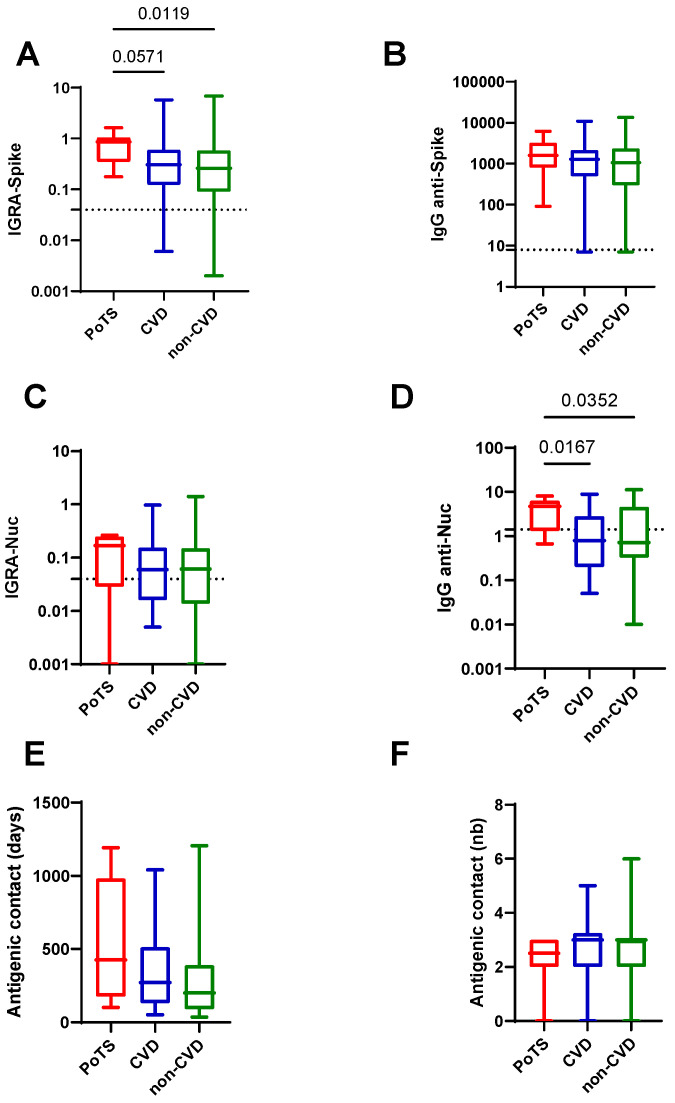
SARS-CoV-2 memory T cell and humoral responses against Spike and nucleocapsid (Nuc) in patients with PoTS (*n* = 8), non-PoTS cardiovascular diseases (CVD; *n* = 26), and non-CVD long COVID patients (*n* = 72). (**A**) Interferon gamma release assay against Spike (IGRA-Spike). (**B**) IGRA-Nuc. (**C**) IgG anti-Spike antibodies. (**D**) IgG anti-Nuc antibodies. (**E**) Time in days from last antigenic contact. (**F**) Number (Nb) of antigenic contacts. Positive thresholds (dot lines, see Section 2 for values) and *p* values < 0.1 are indicated. Dashed line corresponds to the positivity threshold value.

Finally, patients diagnosed with PoTS were distinguished from other CVD and non-CVD long COVID patients in the cohort revealing that anti-Spike cellular response and IgG anti-Nuc humoral response were higher than in other patient groups (Figure 2A–D). This was not explained by a shorter time since the last identified antigenic contact, or the number of antigenic contacts (Figure 2E,F).

## 4. Discussion

The primary objective of this study is to better assess the characteristics of patients with long COVID-associated CVD compared to patients with long COVID symptoms without CVD. The main results from this retrospective cross-sectional study are as follows: (i) specific characteristics included a younger age (below 45 years, mean and median age), to be non-obese, suffering from hyperventilation syndrome, and a clinical background of allergy (i.e., history of urticaria, asthma, allergic rhinitis…); (ii) CVD is associated with the number of identified contacts with the Spike antigen, due to infection and/or vaccination; and (iii) we failed to identify an association with inflammation and common humoral autoimmunity. Of note, the presence of CVD was not associated with a shorter time elapsed since first or last antigenic contact. This is important to consider as patients can have transient CVD after antigenic coronavirus challenge. This also suggests that our patients had a longstanding CVD, and indeed it had in general not been diagnosed before consultation in our center.

Our study shows that some known risk factors for long COVID are also clearly associated with post-COVID CVD [12,13,14,15], such as younger age and HVS status. These factors have also been reported in long COVID PoTS cases [16,17]. Briefly, hyperventilation (HV) causes hypocapnia, sympathetic stimulation with tachycardia, increased systemic vascular resistance including brain vasoconstriction and reduced brain blood flow. Therefore, HV may be a trigger of POTS [18,19,20]. HV-induced hypocapnia also has effects on neurocognitive functions [21]. Conversely, orthostatic cerebral blood flow reduction can also be the initial trigger of tachycardia and hyperventilation in patients with POTS [19]. Another potential confounder may be deconditioning, as it is very common in long COVID patients and may contribute to promoting orthostatic tachycardia [22]. Overall, our study clearly confirms that the association between HVS and dysautonomia holds true in long COVID patients.

On the contrary, obesity rather than a low BMI was associated with long COVID [23] when post-COVID CVD was associated with a lower BMI in the present study. This is an unexpected result, as an experiment showed that a high sympathetic activity correlates with a high share of adipose tissue in young healthy persons [24], and loss of body weight leads to reduced sympathetic activity [25]. However, the proportion of lean and adipose tissue was not determined in our patients.

Finally, some factors do not differ between CVD and non-CVD patients, such as female sex and unvaccinated status. Though vaccination against COVID-19 has been shown to reduce the risk of developing long COVID [26], 16% of participants in our study had not been vaccinated, which is close to the 20.7% reported in a French cohort of 28 million individuals followed for 4 years to test COVID-19 vaccination safety [27]. Vaccinated patients declared to have predominantly received the Pfizer vaccine (71%) as reported in France. But many patients could have received the two types of vaccine when they received multiple doses and the vaccine type as declared by the patient was not associated with the risk of CVD in logistic regression (*p* = 0.67). Therefore, our data should not be interpreted as an increased risk of complication after Pfizer vaccination.

Regarding the roles of allergy and asthma as risk factors for long COVID, meta-analyses have yielded heterogeneous findings. No association has been found with elevated tryptase levels [13,28]. In our cohort, very few patients had elevated tryptase and we found no significant association between CVD and a history of allergy or tryptase level. However, the power of our study may be too low to highlight allergy as a factor associated with CVD. Of note, an elevated basophil count was frequent in the whole cohort and higher levels were associated with CVD. The role of basophils in acute SARS-CoV-2 infection has been reviewed by Miyake and coll. Basophils can be activated by the virus in culture, secreting IL-4 and IL-13, and lower levels of basophils would indicate a poor prognosis [29]. Meirman and Coll showed that basophil count decreases at the time of acute SARS-CoV-2 infection, and then slowly increases but remains lower than in a large control population in the year following acute SARS-CoV-2 infection [30]. More recently, authors reported higher levels of basophils in long COVID Korean patients with chronic cough [31]. Therefore, our data are intriguing. Are basophils a marker of long COVID? This has to be investigated in other cohorts of long COVID patients. The hypothesis of basophils-mediated inflammatory pathways in CVD-related long COVID warrants further investigations.

Multiple lines of evidence implicate the circulating Spike protein and/or the SARS-CoV-2 virus in the pathogenesis of long COVID. First, circulating Spike proteins and viral RNA have been detected in approximately one-third of patients with long COVID up to one year following acute SARS-CoV-2 infection [32,33]. Spike persistence is hypothesized to result from low-level ongoing viral replication [34,35]. Second, rare cases of long COVID manifesting with CVD and PoTS symptoms persisting for months to years following Spike protein vaccination have been documented [36]. This phenomenon may be exacerbated by repeated antigenic exposures, as suggested by the observed association between CVD and the number of expositions in our study. Notably, in our study, no such association was identified when considering either the number of infections (ranging from 1 to 5) or vaccinations (ranging from 0 to 6) separately, which supports a potential role of Spike instead of a direct effect of the virus.

However, such an interpretation needs to be qualified and may be confounded by healthcare access, behavior, exposure risk or other unknown confounders. Though most patients had been treated for acute COVID infection on an outpatient basis and the patients had no lung fibrosis secondary to coronavirus infection, the severity and sequence exposure to SARS-CoV-2 and vaccine were not considered and retrospective data are subject to recall bias. Further studies with many patients would be necessary to document the role of these potential confounders.

Third, although immune evasion has been proposed, our findings indicate that long COVID patients maintain both cellular and humoral immunity against Spike proteins (89–95%) and Nuc proteins (47–68%). Moreover, individuals with PoTS exhibited heightened cellular responses to Spike as well as increased levels of IgG antibodies against the Nuc protein, further arguing against impaired immune control over SARS-CoV-2. Fourth, given that angiotensin-converting enzyme 2 (ACE2) knockdown in murine models is associated with CVD, it is plausible that chronic interactions between the ACE2 receptor and its ligand, the Spike protein, contribute to disease pathology during prolonged exposure [37,38].

A state of low-grade inflammation, characterized by mild elevations in NLR at 23.4% and CRP at 10.4%, was observed in our cohort, despite pro-inflammatory cytokine levels remaining within normal ranges. This finding contrasts with earlier reports describing chronic inflammation marked by higher levels of IL-6, CRP, and NLR in long COVID patients compared to controls [39,40,41]. The primary explanation for this discrepancy is the longer interval since the last immunization, and acute COVID-19 infection, in our study population relative to previous studies, with a median duration of seven months since the most recent identified antigenic exposure. Together with the absence of significant differences between CVD and non-CVD patients, these results suggest that inflammation plays a limited role in the cardiovascular symptoms observed in this context.

How may autoantibodies contribute to CVD in long COVID? To address this question, we examined an extensive panel of common autoantibodies associated with systemic diseases (including ANA, ENA, anti-dsDNA/Chr), antiphospholipid syndrome (anti-CL/β2GPI), as well as central and peripheral nervous system autoimmune disorders. The findings indicate that the autoantibodies tested do not serve as distinguishing markers for long COVID or CVD, although some may be present during acute COVID-19 infection [42,43]. The occurrence of routine ANA, anti-CL/β2GPI, and anti-nervous system autoantibodies in long COVID remains controversial and is partially attributable to variations in study design factors such as the timing post-acute infection, utilization of in-house versus commercial assays, and reliance on self-reported versus clinically confirmed long COVID cases [44]. Concerning symptom associations within long COVID cohorts, fatigue and dyspnea were linked to the presence of anti-U1 snRNP and anti-SSB antibodies, with four cases identified in our investigation and in agreement with previous reports [45]. Although not assessed in this study and with important heterogeneity between reported studies, the most discriminative autoantibodies related to long COVID are those targeting G-protein-coupled receptors, which have been associated with PoTS and gastrointestinal manifestations [46,47]. Additionally, antibodies against interferons and chemokines have been reported in long COVID patients with inconsistent findings; however, to our knowledge, none of these rare antibodies have demonstrated associations with CVD [48,49,50]. At last, we did not search antibodies directed against ganglionic acetylcholine receptor or fibroblast growth factor receptor 3 (FGFR-3) [51,52]. However, none of our patient had a clinically defined ganglionopathy or a small fiber neuronopathy, as these conditions were exclusion criteria, and the causal link between these autoantibodies and dysautonomia is still under debate. Cellular autoimmunity against neural antigens may also play a role. Therefore, our results do not exclude the potential role of humoral or cellular autoimmunity in the occurrence of CVD.

## 5. Conclusions

In conclusion, our results support the role of multiple determinants underlying CVD in long COVID patients. The hypothetical involvement of immunity against the Spike protein and the role of basophils in CVD warrants further research. Our results also underscore the need for identifying novel biomarkers.

## Figures and Tables

**Table 1:** Univariate and multivariate logistic regression analysis describing the risk factors of cardiovascular dysautonomia (CVD) among 34 patients within a cohort of 106 long COVID. Abbreviations: OR: odds ratio; CI: confidence interval; BMI: body mass index; Nuc: nucleocapsid; Ag: antigenic; BMI: body mass index; IGRA: interferon gamma release assay.

Variable	Univariate OR [95% CI]	*p* Value	Multivariate OR [95% CI]	*p* Value
Sexe		0.52		
Age < 45	2.42 [1.04–5.64]	0.04	2.9 [1.09–7.77]	0.03
BMI	0.9 [0.82–0.98]	0.02	0.87 [0.79–0.97]	0.01
Hyperventilation syndrome	1.99 [0.87–4.55]	0.10	2.9 [1.10–7.55]	0.03
Allergic background		0.12		
Basophil count ≥ 0.06 G/L	2.09 [0.89–4.91]	0.09	3.26 [1.9–8.8]	0.02
Infections ≥ 2		0.49		
Vaccination ≥ 3		0.81		
Vaccine type		0.67		
Immune contacts ≥ 3	2.50 [0.78–8.07]	0.12	4.1 [1.10–14.9]	0.03
Time since last antigenic contact >1 year	2.66 [1.13–6.3]	0.02		
IgG anti-Spike		0.64		
IgG anti-Nuc		0.41		
IGRA-Spike		0.57		
IGRA-Nuc		0.87		

**Table 2:** Results of the sensitivity analysis restricted to non-POTS patients.

Variable	Multivariate OR [95% CI]	*p* Value
Age < 45	2.73 [0.93–7.99]	0.07
BMI	0.88 [0.78–0.98]	0.03
Hyperventilation syndrome	2.95 [1.04–8.41]	0.03
Basophil count ≥ 0.06 G/L	4.31 [1.37–13.6]	0.01
Immune contacts ≥ 3	4.01 [0.94–17.1]	0.06

## Data Availability

The datasets generated during the current study are available from the corresponding author on request.

## Abbreviations

The following abbreviations are used in this manuscript:

CVD	cardiovascular dysautonomia
HVS	hyperventilation syndrome
IGRA	interferon gamma release assay
POTS	postural orthostatic tachycardia syndrome
